# Impact of clinical supervision on field training of nursing students at Urmia University of Medical Sciences

**Published:** 2016-04

**Authors:** MOHAMMADREZA DEHGHANI, SHIRIN GHANAVATI, BEHROUZ SOLTANi, NADER AGHAKHANI, SEZANEH HAGHPANAH

**Affiliations:** 1 Quality Improvement in Clinical Education Research Center, Education Development Center, Shiraz University of Medical Sciences, Shiraz, Iran;; 2 Kermanshah University of Medical Sciences, Kermanshah, Iran;; 3 Inpatient Safety Research Center, Urmia University of Medical Sciences, Urmia, Iran;; 4 Haematology Research Center, Shiraz University of Medical Sciences, Shiraz, Iran

**Keywords:** Clinical, Nursing, Training, Students

## Abstract

**Introduction:**

Obtaining clinical competency in clinical education is one of the problems in nursing and use of the new methods of clinical training is very important. Clinical supervision is one of the methods used as a mechanism to promote knowledge and skill for promoting professional performance in nursing students. This study is carried out to determine the impact of clinical supervision on field training of nursing students at Urmia University of Medical Sciences.

**Methods:**

In the present experimental study, 32 nursing students were enrolled in the study based on census and randomly assigned into two groups of experimental and control by block randomization. Clinical supervision was used in the experimental group and the control group received routine clinical trainings in the field. The students’ clinical skills were assessed using a researcher-made checklist, the validity of which was confirmed through content validity method by 13 faculty members and its reliability was approved by test-retest method on 20 nursing students in the form of a pilot study and through Cronbach’s alpha (87%). Data were analyzed using SPSS, version 14.

**Results:**

‍There was a significant difference between the experimental and control groups in clinical skills such as recognition and administration of medication, team participation,  patients and their relatives’ education, considering the safety,  infection prevention and  nursing process (p<0.005).

**Conclusion:**

The study demonstrated that in clinical supervision process, students have a better communication and cooperation with their instructor and with each other and their confidence and understanding and the amount of learning in practical skills was enhanced more than routine clinical training. The implementation of this clinical training method for students of nursing and other fields of medical sciences is recommendable.

## Introduction

During the last decades, nursing education has had many changes because of movement from practical training to academic education. These changes have made many challenges for all parties involved in the nursing education field (1). On the other hand, reports about insufficient support to nursing students are frequent when they want to use theory in practice and vice versa (2); also, the viewpoints of nursing students on clinical education and supervision have been mentioned in various studies. They have stressed the importance of being involved in patient care during clinical education (3, 4), of experiences of responsibility and independence (4), and of being part of a team (3, 5). This is well documented in the literature that a supportive environment in clinical learning is important. Studies such as those of Bourgois et al. (2011) and Gidman et al. (2011) show that asupportive structure like facilitating nurses and clinical lecturers during clinical education is very important to students’ learning process (3, 6); thus, a structured system of communication and feedback must bedeveloped to support and defineclearly the roles and responsibilities of those involved in clinical and academic settings to achieve the goals and learning outcomes of nursing education (7). Learning outcomes are those which the learner should know or be able to demonstrate after the completion of the learning process or after finishing a course (8); also, a constructive clinical learning environment with adequate opportunities for the improvement of confidence and competence with a focus on students’ learning needs is essential (9). If students need to acquire knowledge and skills in clinical training, someone must be there to supervise and demonstrate how theoretical knowledge can be integrated into practice for them (10, 11), in order to achieve this goal and fill the gap between theoretical knowledge and its application in practical setting, many educational supportive models like clinical supervision were developed.

Clinical supervision is a process of supporting and learning which enables the students to develop their knowledge and competence for their own practice, and enhance client protection and safety of care in complex clinical situations (12); by conducting such a supervision, nursing students can acquire more skillful competencies.

Due to lack of studies done in nursing supervision at fields in our country,  this study was carried out to assess the impact of clinical supervision on field training of nursing students at Urmia University of Medical Sciences to be a guide for further researchers and stakeholders. 

## Methods

This is an experimental study assessing the impact of clinical supervision on field training of nursing students at Urmia University of Medical Sciences.

According to the small number of study population, all nursing students of Urmia University of Medical Sciences who attended the infectious disease wards based on census were enrolled in the study and this is a reason that study population and samples are similar in number (n=32). Due to the entrance of the population in the study, there was no need to mention the statistics formula about how the study population was selected. The participants were randomly assigned into two groups of experimental and control by block randomization. The experimental group received clinical supervision in field training and the control group received routine clinical training.

To gather data, a researcher-made checklist was used. The checklist included two parts; the first part consisted of demographic characteristics and the second part was related to assessing cognitive dimensions and practical skills of the participants. The second part of the checklist itself included six parts: recognition and administration of medication, education to patients and their relatives, team participation, infection prevention, considering the safety, and paying attention to nursing process. Validity of the instrument was confirmed through content validity method by 13 faculty members of Urmia University of Medical Sciences.

In order to evaluate the reliability of this checklist, the test-retest method was performed as a pilot study in 20 nursing students (as suggested by a researcher and cooperators in the study designing) using Cronbach’s alpha coefficient. Finally, α=87% was calculated.

The control group passed their course with their routine instructor (as a clinical attending) without involvement of clinical nurses.

In this study, the head nurses of the wards where the study was conducted were selected as clinical supervisors. To prepare clinical supervisors and instruct them about the method of conducting the principles of clinical supervision, three sessions were conducted (2 hours each session).

Clinical supervision phase included three parts, each part lasting for two weeks; each of the three parts began with a supervision session, and these sessions were conducted in the ward setting and lasted for 30 minutes. During all the sessions, each member presented his\her idea and finally, based on study objectives, administrative manners were developed. At the end of each session, all ofthe members documented the session contents. Between the sessions, the clinical supervisor gathered the students’ functions data by using a checklist. Documented items in supervision session checklist were considered as principles of the students’ administrative evaluation.

The study aim was explained to the students by the clinical instructor and their course began under observation of their college instructor and nurses at fields. The nurses as instructors’ assistants were responsible for education and evaluation of students and the clinical instructor was responsible for solving the students and nurses’ problems and supervising their administration. At the end of each course, the students’ clinical skills were evaluated through a checklist by the nurses who acted as clinical instructor assistant.

The present study was approved by the Ethics Committee of Urmia University of Medical Sciences. Students were provided with verbal information about the study objectives and design; then, informed consents were obtained from them. The subjects were also ascertained about the confidentiality and anonymity of their information as well as their right to withdraw from the study. Data were analyzed using SPSS software version 14 through descriptive statistics (frequency, mean, and standard deviation). To compare the scores before and after the intervention in each group, Wilcoxon signed-rank test was done. Comparison of the scores between the two groups before and after the study as well as changes in each group was done by Mann-Whitney test. The significance level was considered less than 0.05. 

## Results

All of the participants were less than 20 years old. Mean age of the participants was 18.06±1.13. 50% of them were female and 96.8% single (Table 1).

**Table 1:** Characteristics of the studied nursing students (n=32)

**Characteristics**	**Values (percentage)**
**Age**	18.06±1.13
**Marital status**	
**Single**	31 (96.875%)
**Married**	1 (3.125%)
**Gender**	
**Female**	16 (50%)
**Male**	16 (50%)

The level of skills and their differences before and after the study conducted in both experimental and control groups are demonstrated in Table 2.

**Table 2:** Comparison of the scores before and after the intervention in both experimental and control groups and between the two groups

**Variables**				**Group**		**p**
				**Experimental ** **N=16**	**Control ** **N=16**	
**Recognition and administration of medication**	**Pre test**			27.90±3.70	39.46±4.70	<0.001*
	**Post test**			40.16±3.87	38.51±3.22	0.199
**Change **				12.26±3.35	-0.95±3.40	<0.001*
**p (within)**				<0.001*	0. 509	----------
**Education to patients and their relatives**	**Pre test**			7.39±2.25	11.45±4.60	0.003
	**Post test**			15.10±2.45	11±2.50	<0.001*
**Change **				7.71±2.20	-0.45±3.55	<0.001*
**p (within)**				<0.001*	0.733	----------
**Team participation**	**Pre test**			8.60±2.35	7.70±1.72	0.226
	**Post test**			17.10±2.00	9.96±2.06	<0.001*
**Change **				8.50±2.21	2.26±1.96	<0.001*
**p (within)**				<0.001*	0.002	----------
**Considering the safety**	**Pre test**			40.03±5.05	47.5±6.13	<0.001*
	**Post test**			66.90±5.59	52.03±3.68	<0.001*
**Change **				26.87±5.35	4.53±4.82	<0.001*
**p (within)**				<0.001*	0.016	----------
**Infection prevention**	**Pre test**			51.92±3.95	52.40±6.93	0.811
	**Post test**			67.34±4.97	59.03±4.00	<0.001*
**Change **				15.42±4.5	6.63±5.8	<0.001*
**p (within)**				<0.001*	0.002	----------
**Paying attention tonursing process**	**Pre test**			31.12±4.15	37.70±6.40	<0.001*
	**Post test**			71.34±5.01	49.03±4.02	<0.001*
**Change **				40.22±4.89	11.33±5.4	<0.001*
**p (within)**				<0.001*	<0.001*	----------

At the end of the current study, the experimental group students stated that clinical supervision had further motivated them to learning.

## Discussion

Results of our study indicated that the experimental group in clinical skills including recognition and administration of medication, education to patients and their relatives, team participation, infection prevention, considering the safety, and paying attention to the nursing process had a significant differences with the control group; thus, these results showed the significant influence of this clinical supervision model on learning outcomes of nursing education.

Kristofferzon et al. in a study done in 2013 have revealed that nursing students seemed to be satisfied with clinical supervision provided by preceptors, teachers, and clinical instructors. Also, they stated that the supervision provided by the group of facilitators helped the students to fulfill their learning outcomes to a large extent (13).

The results of the following study are not consistent with our findings. In a study by Lofmark et al. conducted in 2012, nursing students’ satisfaction with supervision by preceptors and teachers and fulfillment of learning outcomes during clinical practice were investigated. The findings of the study demonstrated that supervision by clinical teachers was rated more highly than supervision by preceptors. Students had filled out a questionnaire at the end of the clinical practice period and they had estimated learning outcomes to be achieved to a high extent. Fulfillment of learning outcomes was even more strongly related to supervision by teachers than by preceptors. The results of their study demonstrated the overall positive benefits of supervision during the clinical education period (14). According to our study findings, in earlier studies, appreciation and benefits of support from preceptors have been shown (15, 16).

The findings of Carveret al.’s study about group clinical supervision in 2014 showed that students had become increasingly more autonomous learners; also, it was revealed the value of such a program in enabling the students to become supervisees in group clinical supervision (12). Lingren et al. evaluated a group supervision model aiming to increase the nursing students’ knowledge, understanding and insights into professional nursing. The findings showed that group supervision had provided important support in these areas and almost all students wanted to participate in group supervision in the future (17). The results of a study conducted about clinical supervision and complementary therapists have revealed that the clinical supervision was valued by the participants in managing the challenges complementary therapists face in their work environment (18).

In the most recent studies, the application and effect of clinical supervision has been assessed but in the following mentioned study researchers have assessed the perception of lecturers about clinical supervision in students. Lingren and Althin (2010) conducted a study entitled “nurse lecturers’ perceptions of what baccalaureate nursing students could gain from clinical group supervision”. Their study showed that the nurse teachers felt that their students had become aware of their own strengths and weaknesses by clarifying their thoughts in the session; also, they felt that the supervision sessions could increase the students’ personal and professional strength by widening their understanding both cognitively and emotionally. Their findings have shown that the students’ awareness of the theory practice gap and the necessity to continue searching for new knowledge was considered to increase during the supervision program; also, the students’ self-esteem and self-confidence could be strengthened through the confirmation and acknowledgment they received from the supervisor (19).

Paying attention to the above-mentioned studies demonstrate the positive effect of different clinical supervision models on instruction process based on both teachers and students viewpoint. 

## Conclusion

Based on the present results, in clinical supervision process, learners have a better communication and cooperation with each other. Their confidence and understanding will increase and the amount of learning in practical skills is more than the routine clinical training. The implementation of this method in this form for the students of nursing and other fields of medical sciences is recommendable.

**Limitations**

The present study had some limitations. The first limitation of this study was that the students were selected from one university of one of the big cities of Iran. Consequently, the results cannot be generalized to the students studying in other universities of the country. The second limitation was the small sample size which limited the power of the study to detect the statistically significant differences. Of course, by increase the sample size, the possibility of a markedly deviant sample diminishes.

**Recommendation**

The results of the present study could not be generalized to all students of different universities with different cultured due to its small group of participants. Therefore, further studies are required in this regard. 

## References

[1] Barett D (2007). The clinical role of nurse lecturers: past, prese‎nt and future. Nurse Education Today.

[2] Henderson A, Fox R, Malko-Nyhan K (2006). An evaluation of preceptors’ perceptions of educational preparation and organizational support for their role. Journal of Continuing Education in Nursing.

[3] Gidman J, McIntosch A, Melling K, Smith D (2011). Students' perceptions of support in practice. Nurse Education in Practice.

[4] Hellström-Hyson E, Mårtensson G, Kristofferzon ML (2012). To take responsibility or to be an onlooker. Nursing students' experience of two models of supervision. Nurse Education Today.

[5] Bradbury-Jones C, Sambrook S, Irvine F (2011). Empowerment and being valued: a phenomenological study of nursing students' experiences of clinical practice. Nurse Education Today.

[6] Bourgeois S, Drayton N, Brown AM (2011). An innovative model of supportive clinical teaching and learning for undergraduate nursing students: a cluster model. Nurse Education in Practice.

[7] Andrews GJ, Brodie DA, Andrews JP, Hillan E, Gail Thomas B, Wong J (2006). Professional roles and communications in clinical placements: a qualitative study of nursing students' perceptions and some models for practice. International  Journal of Nursing  Studies.

[8] Zabalugui A, Macia L, Marquez J, Ricomá R, Nuin C, Mariscal I (2006). Changes in nursing education in the European Union. Journal of Nursing Scholarship.

[9] Croxon L, Maginnis C (2009). Evaluation of teaching models for nursing practice. Nurse Education in Practice.

[10] Lambert V, Glacken M (2005). Clinical education facilitators: a literature review. Journal of  Clinical Nursing.

[11] Lambert V, Glacken M (2004). Clinical support roles: a review of the literature. Nurse Education in Practice.

[12] Carver N, Clibbens N, Ashmore R, Sheldon J (2014). Mental health pre-registration nursing students’ experiences of group clinical supervision: A UK longitudinal qualitative study. Nurse Education in Practice.

[13] Kristofferzon ML, Mårtensson G, Mamhidir AG, Löfmark A (2013). Nursing students' perceptions of clinical supervision: The contributions of preceptors, head preceptors and clinical lecturers. Nurse Education Today.

[14] Anna Löfmark A, Thorkildsen K, Råholm M, Natvig GK (2012). Nursing students’ satisfaction with supervision from preceptors and teachers during clinical practice. Nurse Education in Practice.

[15] Papp I, Markkanen M, Bonsdorff M (2003). Clinical environment as a learning environment: student nurses’ perceptions concerning clinical learning experiences. Nurse Education Today.

[16] Billay D, Myrick F (2008). Preceptorship: an integrative review of the literature. Nurse Education in Practice.

[17] Lindgren B, Brulin C, Holmlund K, Athlin E (2005). Nursing student perception of group supervision during clinical training. Journal of Clinical Nursing.

[18] Mackereth PA, Parkin S, Donald G, Antcliffe N (2010). Clinical supervision and complementary therapists: An exploration of the rewards and challenges of cancer care. Complementary Therapies in Clinical Practice.

[19] Lindgren B, Athlin E (2010). Nurse lecturers’ perceptions of what baccalaureate nursing students could gain from clinical group supervision. Nurse Education Today.

